# Variation in echolocation call emission of Neotropical insect-eating bats in response to shifting ambient temperatures

**DOI:** 10.1242/jeb.251076

**Published:** 2025-10-24

**Authors:** Paula M. Iturralde-Pólit, Marcelo Araya-Salas, Holger R. Goerlitz, Gloriana Chaverri

**Affiliations:** ^1^ Sede del Sur, Universidad de Costa Rica, Golfito 60701, Costa Rica; ^2^ Acoustic and Functional Ecology, Max Planck Institute for Biological Intelligence, 82319 Seewiesen, Germany; ^3^ Smithsonian Tropical Research Institute, Balboa 0843-03092, Ancón, Republica de Panamá

**Keywords:** Atmospheric attenuation, Detection distance, Behavioural plasticity, Climate change, Vocal flexibility, Insectivorous bats

## Abstract

The sensory systems of animals are essential for them to respond to environmental cues and signals. However, their functionality might be altered by climate change. Most bats, for example, rely on acoustic signal emission to acquire food, but their high-frequency echolocation calls are strongly attenuated in the air. Attenuation in air changes with changing weather conditions, which can lead to shifts in echo-based prey detection distance. However, bats can adjust call parameters to the task and environment, and this behavioural plasticity may help them to counteract potential increases in sound attenuation to keep echo detectability constant. We explored this ability in a community of insectivorous bats in a montane forest of Costa Rica. We recorded bat echolocation calls in response to experimentally increased temperatures, simulating intermediate and arguably realistic projected climate change scenarios. We calculated atmospheric attenuation and detection distance for each temperature and echolocation call. We found some changes in source level and call duration, yet not in peak frequency, and responses to increasing atmospheric attenuation were not consistent across species. This might be explained by several non-mutually exclusive reasons, including that the experimental increase in temperature and change of atmospheric attenuation were not sufficient to affect close-range prey detection. Ultimately, this study contributes to our understanding of sensory system adaptation under the pressure imposed by climate change.

## Teaser

**Highlighted Article:** Studying acoustic adjustments of insectivorous bat echolocation calls under projected climate change conditions reveals intra-specific variability, with consequences for prey detection.

## INTRODUCTION

Climate change is affecting biodiversity in various ways; however, most research has been mainly focused on assessing shifts in potential distributions of local and invasive species (Anderson, 2013; Crespo–Pérez et al., 2015; Gallardo et al., 2017; Iwamura et al., 2020; Pecl et al., 2017; Peterson et al., 2018), species phenology (Parmesan, 2006) and species vulnerability (Foden et al., 2013; Rahbari et al., 2017). In addition, available data are geographically biased (Martin et al., 2012; Meyer et al., 2015), precluding us from accurately predicting its effect more broadly. Many climate change studies reference global response patterns, yet latitudes below 30° remain underrepresented (Feeley et al., 2017) despite being the areas with the greatest biodiversity (Barlow et al., 2018). Furthermore, those studies often forecast shifts based on correlations of available information, but they rarely consider the mechanisms that shape species' responses to increasingly warming conditions (Urban et al., 2016). This can only be assessed with experimental studies, which are crucial in determining the ability of species to respond to climate change (Razgour et al., 2019; Willis et al., 2015). For example, understanding potential changes in a species' perceptual abilities under changing conditions provides insights into its ability to overcome environmental shifts, but these studies are still scarce in tropical regions (Beever et al., 2017).

Sensory ecology studies how organisms acquire and respond to environmental cues and signals. Animals rely on this information for courtship, individual recognition, orientation and prey detection, among others (Stevens, 2013). Unfortunately, the functionality of animal sensory systems may be altered by several factors; for example, by noise pollution in both terrestrial (Dominoni et al., 2020; Tuomainen and Candolin, 2011) and aquatic environments (Johnson et al., 2009; Kelley et al., 2018). Most recently, although data are still scarce, several studies suggest that anthropogenic climate change may be an additional factor that affects animals' sensory perception. For example, in aquatic environments, higher CO_2_ concentrations and ocean acidification can impair olfactory sensitivity in fish, reduce the efficiency of visual cues that indicate predator presence and disrupt auditory-guided behaviours (Draper and Weissburg, 2019; Kelley et al., 2018; Porteus et al., 2018; Rivest et al., 2019). Other effects of climate change, such as changing temperatures, could affect terrestrial animals. For instance, lizards, moths and flies could suffer a reduction in the effectiveness of chemical sexual signals, which are essential for mate choice (Groot and Zizzari, 2019; Martín and López, 2013).

Sound is a central stimulus for many animals to acquire environmental information (Dusenbery, 2001). For example, echolocation is a sensory mechanism by which animals produce high-frequency vocal signals and listen to the returning echoes to perceive their surroundings. Echolocating animals, such as bats, use this mechanism for spatial orientation and often to detect, localize and intercept prey (Schnitzler and Kalko, 2001). Bats exhibit remarkable flexibility in adjusting their signals based on behavioural tasks and habitat conditions (Barclay, et al 1999; Neuweiler, 1989; Schnitzler et al., 2003). For instance, bats increase call duration and lower call frequency when moving from near-ground cluttered environments to uncluttered forest canopy (Gillam et al., 2009). Bats also modify call structure to reduce masking effects and discriminate echo delays depending on the distance at which they localize a target (Denzinger and Schnitzler, 2013; Moss and Schnitzler, 1989) or according to habitat conditions (Kalko and Schnitzler, 1993).

The emitted calls of echolocating bats may also be influenced by weather conditions, as temperature and humidity play a significant role in the atmospheric attenuation of the high-frequency calls commonly used by these mammals (Goerlitz, 2018). As temperature increases, absorption can either increase or decrease, depending on both the frequency of the call and the relative humidity (RH) of the environment (Goerlitz, 2018). For example, high-frequency calls (>80 kHz) generally experience greater absorption with rising temperatures, especially under low to medium RH (30–60%). However, when RH is high (90%), absorption may decrease even as temperature increases. In contrast, for low-frequency calls (<50 kHz), temperature has a strong positive effect on absorption only when RH is low, while at high RH, temperature appears to have little to no effect. These complex interactions highlight that the relationship between climatic conditions and sound absorption is highly context dependent and cannot be easily generalized across species or environments. Given these constraints, animals such as bats that rely on acoustic signals may need to adjust their vocalizations to maintain effective communication and foraging. Adjustments in call structure may help mitigate sound attenuation (Römer, 2001), but the specific combination of temperature, humidity and signal frequency shapes their effectiveness (Luo et al., 2014; Obrist, 1995; Snell-Rood, 2012; Surlykke and Kalko, 2008). Therefore, understanding how bats optimize sound propagation and maintain foraging efficiency requires consideration of local environmental conditions.

Changing weather conditions, originating from either short-term spatio-temporal variation or long-term climate change, affect the volume of space over which bats can detect prey (Luo et al., 2014). Hence, bats may rely on vocal plasticity to compensate for this effect. While some species show long-term acoustic signal divergence associated with adaptation of sensory systems to local environmental conditions (geographical variation in average weather parameters; Chen et al., 2009; Maluleke et al., 2017; Mutumi et al., 2016), individuals can also plasticly adjust their signals in response to seasonal and daily weather fluctuations (Chaverri and Quirós, 2017; Snell-Rood, 2012). These findings show that bats have control over their acoustic signals, making them a good study system to investigate adjustments in vocal production in response to the predicted shifts in weather due to climate change.

To date, studies focusing on the sensory responses of bats to fluctuations in atmospheric conditions on a short temporal scale are scarce. Some studies have assessed effects on detection distance (de Framond et al., 2023b) and adjustments in echolocation calls in a temperate region (Snell-Rood, 2012). To our knowledge, only one study has evaluated the association between weather conditions and acoustic parameters of calls in echolocating Neotropical bats (Chaverri and Quirós, 2017). Neotropical species, particularly those in higher elevations, have received little attention despite living in a region predicted to experience substantial changes in weather (Boehmer, 2011; Still et al., 1999) and significantly higher maximum temperatures (Enquist, 2002). In addition, the more constant weather conditions in the tropics compared with temperate regions contribute to narrower thermal tolerances, smaller distribution ranges, higher species turnover along altitudinal gradients (Ghalambor et al., 2006) and higher levels of endemism (Chaverri et al., 2016). Altogether, these factors could render tropical bat species more sensitive and vulnerable to climate change, posing a potential threat to their long-term survival.

Here, we aimed to gather empirical data on the acoustic responses of a community of Neotropical insectivorous bats to changes in the abiotic environmental conditions known to affect sound transmission. We hypothesized that echolocating bats modify their call parameters in response to changing atmospheric conditions, which affect sound attenuation and, thus, maximum detection distance. In response to increasing atmospheric attenuation, bats might decrease call frequency, increase emitted call level, increase call duration, or any combination thereof in such a way as to maintain maximum detection distance (Luo et al., 2014; Snell-Rood, 2012). We simulated increasing temperatures in line with climate change scenarios and recorded the bats' echolocation calls, analysed their call parameters, and calculated the resulting atmospheric attenuation and maximum detection distance to assess the influence of atmospheric attenuation on call emission. Because environmental conditions are often highly variable, assessing vocal flexibility in bats at short to medium temporal scales will help predict species’ sensitivity and resilience to some of the abrupt shifts in weather conditions caused by climate change (Alley et al., 2003).

## MATERIALS AND METHODS

### Field site and species

We collected data at Las Cruces Research Station located in Southern Costa Rica at Coto-Brus county, close to the boundary of the largest protected area in the country (Parque Internacional La Amistad). The site includes tropical pre-montane and lower montane forests with frequent presence of clouds (Enquist, 2002). Altitude ranges from 1200 to 1800 m above sea level, ambient temperature between 15°C at night and 28°C during the day, and relative humidity fluctuates between 60% and 100% throughout the year. These ecosystems are crucial for biodiversity conservation because of the high species diversity and endemism (Cadena et al., 2012), where bats are no exception (Chaverri et al., 2016; Pineda-Lizano and Chaverri, 2022). However, it has been predicted that the Neotropical region will suffer significant climate change-induced shifts in weather conditions of up to 4°C with slight differences between the wet and dry seasons (Karmalkar et al., 2011) and that highland sites may suffer some of the most significant effects on biodiversity (Karmalkar et al., 2008; Mata-Guel et al., 2023).

We focused on insectivorous bats from the genera *Myotis* and *Eptesicus* (family Vespertilionidae). These bats often fly in the understory (Kalko et al., 2008) and are mainly edge-space foragers, yet also move between different types of habitats when searching for prey, thereby adapting their echolocation calls to different foraging situations (Denzinger and Schnitzler, 2013).

We captured bats with mist-nets from 17:45 h (before dusk at the study site), when vespertilionid bats started their activities, until 21:30–22:00 h, when the first bout of bat activity dropped. We aged bats as young and adults based on the degree of ossification of the metacarpal–phalange joints (Brunet-Rossinni and Wilkinson, 2009). Then, we sexed and taxonomically identified all individuals, fed them with mealworms (larvae of *Tenebrio molitor*), provided water *ad libitum*, and kept them individually in cloth bags until the start of the experiments (around 22:30–23:00 h).

Taxonomic identification of species in the field can be difficult, especially when species are cryptic, as is the case with some *Myotis* species (York et al., 2019). As genetic identification was not possible, we followed the Field Key to the Bats of Costa Rica and Nicaragua (York et al., 2019) for identification. We identified *Myotis oxyotus* and *Myotis riparius* based on the arrangement of the first two premolars. For *Myotis pilosatibialis*, we verified that the uropatagium and legs had fur at least to the knee. Identification of *Myotis nigricans* and *Myotis elegans* was more problematic, and we collected few individuals of these species because they are more common in the lowlands. To avoid misidentification, we grouped these individuals (herein *Myotis nigricans*/*elegans*) for further analyses.

We conducted this study in accordance with the guidelines for the capture and handling of wild mammals for research established in Sikes (2016). We followed ethical standards for animal welfare of the Costa Rican Ministry of Environment and Energy, Sistema Nacional de Áreas de Conservación, permit no. R-SINAC-ACLAP-162-2017. Protocols were also approved by the University of Costa Rica's Institutional Animal Care and Use Committee (CICUA-27-2015).

### Experimental setup

We recorded the echolocation calls of individual free-flying bats in an outdoor flight cage (6×2.5×2.5 m^3^) with a four-microphone array. The cage walls were made of synthetic cloth, reducing the exchange of air between the cage and the environment. To reduce reverberations, the cage walls and ground were covered with sound-absorbing fabric, with additional sound-absorbing foam on the ground.

**Fig. 1.**
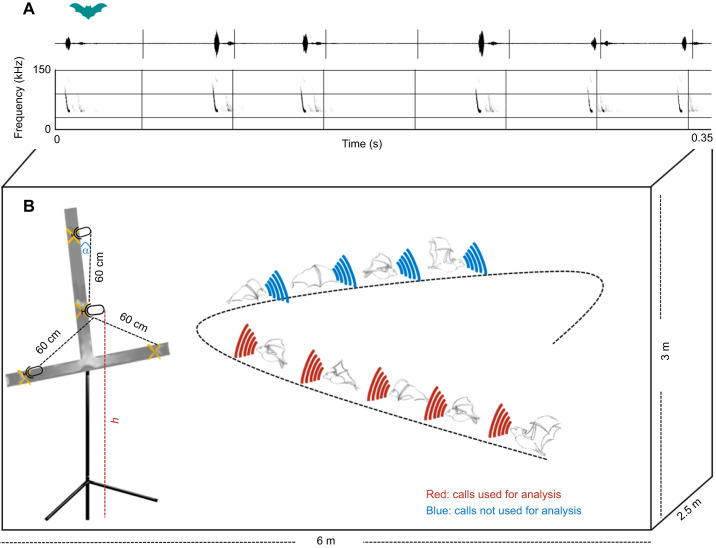
**Experimental setup.** (A) Example of a sequence of calls selected for analysis. (B) Bats flew freely and individually inside the outdoor flight cage. Their calls were recorded using a four-microphone array placed on one side of the flight cage. The microphones were arranged in a symmetrical star shape with three peripheral microphones separated by 60 cm around a central microphone, mounted on a T-shaped structure, where α is the angle relative to the vertical axis and *h* is the height of the microphone. For analysis, only calls emitted by bats flying towards the microphone array (indicated in red) were considered, while calls emitted when bats were flying away from the array (indicated in blue) were excluded.

We positioned the microphone array centrally at one side of the flight room to record the echolocation calls of the bats (Fig. 1A, example of calls recorded from *M. pilosatibialis*). The array consisted of four omnidirectional electret ultrasound microphones (Knowles FG-O, Avisoft Bioacoustics, Glienicke/Nordbahn, Germany) arranged in a symmetrical star-shape and mounted on a T-shaped metal structure covered with sound-absorbing foam. The three outer microphones were positioned at a distance of 60 cm from the central microphone (Fig. 1B). The height and angle of the central microphone were measured every recording session to ensure precise positioning. Microphone signals were recorded with an Avisoft Ultra Sound Gate 416 H and Avisoft Recorder software to a four-channel WAV file at 500 kHz sampling rate and 16-bit amplitude resolution and maximum gain without clipping the calls.

We calibrated the frequency response and the directionality of the central microphone, which was used for call analysis, before the experiment. We played pure tones with constant frequency from 5 to 95 kHz in steps of 5 kHz from a loudspeaker (Vifa, Avisoft Bioacoustics) and recorded them with a measuring microphone with flat frequency response (GRAS Sound and Vibration A/S, Holte, Denmark) placed at 50 cm distance. We recorded the same pure tones with the central microphone from directions from 0 to 90 deg in 5 deg steps. By comparing recordings on the measuring microphone and the central microphone, we obtained the frequency response and directionality of the central microphone, which we used to correct each recorded call to obtain the call as arriving at the microphone, which was further corrected to obtain the call as emitted by the bat (see ‘Estimating call parameters’ below).

### Recording procedure

To investigate whether and how increasing temperature affects call parameters, we recorded the echolocation calls of individual free-flying bats under three different temperature conditions. The least and most extreme climate change projections (IPCC, 2023) predict an increase in the ambient temperature of 1.4°C (SSP1-1.9) and 4.4°C (SSP5-8.5), respectively. We aimed to simulate the intermediate and arguably more realistic scenarios SSP2-4.5 and SSP3-7.0, which predict temperature increases of 2°C and 4°C.

We tested each bat for one night by presenting three different conditions: current ambient temperature *T*_a_, *T*_a_+2°C and *T*_a_+4°C, with average RH of 94%, 94% and 89%, respectively. First, we recorded each bat under unmodified ambient atmospheric conditions (*T*_a_), in which the temperature and humidity of the flight cage were similar to external conditions. For the two subsequent trials, we increased the temperature in the flight cage by 2°C and 4°C, respectively, using two electric heaters placed in the middle of the flight cage. Once we reached the target temperature, we removed the heaters from the cage to reduce reverberations. Temperature and RH in the flight cage were continuously recorded with two weather loggers (Kestrel 4000, pocket weather tracker, KestrelMeters, Boothwyn, PA, USA), one hanging from the T-shaped structure and the second in the middle of the flight tent to increase the likelihood of achieving a representative temperature measurement. Each bat was tested under all three conditions and in the same sequence. If more than one bat was captured in one night, we first tested all of them individually under *T*_a_, and then under the sequentially increased temperatures.

For each of the three trials, we released the bat into the flight cage for no longer than 5 min and recorded between five and ten audio files of approximately 10 s each. After each trial, we caught the individual with a hand net, fed it with mealworms and provided water *ad libitum*. After the experiments, the bats were released at their capture site.

### Estimating call parameters

Analyses were not conducted blind to treatment, as the same research team carried out both the recordings and the acoustic parameter extraction. However, parameter extraction was performed using automated software, ensuring objective and reproducible results independent of treatment conditions. We checked all recordings using SASLab Pro (Avisoft Bioacoustics) and manually determined the time and location of at least four consecutive calls per recording. For each call, we estimated acoustic parameters (maximum and minimum call frequency, call duration and inter-call interval), which were then averaged across calls per species. We used this information to determine an appropriate bandpass filter for every recording session for final call analysis. We used the custom-developed TOAD Suite software package (de Framond et al., 2023a,b; Hügel et al., 2017; Lewanzik and Goerlitz, 2018) for MATLAB (version R2007b; The MathWorks, Inc., Natick, MA, USA) to calculate the bats' spatial position for each emitted signal based on the time-of-arrival differences (TOAD) from the central to the outer microphones of the array (Koblitz, 2018) and the speed of sound for the current air temperature and relative humidity.

We then reconstructed the 3D flight trajectories and manually selected at least four consecutive calls without overlapping echoes (quality calls) from trajectory segments where the bats were flying towards the microphone array. All selected calls were automatically corrected for atmospheric absorption and spherical spreading on the way from the bat to the microphone, and the frequency response and directionality of the microphone, to obtain the call as emitted by the bat at 10 cm from its mouth. We then automatically calculated call duration based on the smoothed Hilbert envelope at −12 dB relative to the envelope's peak amplitude value; peak frequency (the frequency with the highest amplitude); and apparent source level (aSL) as the root mean square (rms) relative to 20 µPa and at 10 cm to the bat's mouth (rms dB re. 20 µPa at 10 cm). As bat calls are highly directional and not necessarily emitted towards the microphone, the aSL is an underestimation of the real on-axis source level (SL).

We excluded calls with signal-to-noise ratios <30 dB and those in which the maximum energy was detected in the second harmonic. This resulted in a dataset of 5104 calls of five species (groups): *M. nigricans*/*elegans* group: 735 calls; *M. pilosatibialis*: 2567; *M. riparius*: 978; *M. oxyotus*: 331, *Eptesicus brasiliensis*: 493.

To approximate the real SL from aSL, we only kept calls above the 90th percentile of the aSL within one experimental trial of one bat. By filtering out calls below this threshold, we also aimed to minimize the impact of off-axis emission on peak frequency, as high-frequency components are more susceptible to off-axis attenuation. This resulted in a total of 1002 calls for final analysis (Table 1). The most abundant species in our sample was *M. pilosatibialis*, with 31 individuals, from which we also recorded the highest number of calls in the final dataset (494). We obtained a mean (±s.d.) of 6±3 calls per individual per experimental temperature.

**Table 1.** The number of bat individuals and echolocation calls recorded at each experimental temperature for each species

Species (no. of individuals)	Temp. (°C)	No. calls	Variable	Peak frequency				Source level				Duration			
				M1		M2		M1		M2		M1		M2	
				Est.	*P*	Est.	*P*	Est.	*P*	Est.	*P*	Est.	*P*	Est.	*P*
*M. pilosatibialis* (*n*=31)	*T*_a_	146	Temp.	−0.22	0.258	−1.284	**0.004**	0.10	0.358	−0.54	0.094	0.05	**0.002**	0.05	0.094
	+2°C	193	Dist. to mic.			−12.51	**0.006**			−5.96	0.080			0.17	0.590
	+4°C	155	Temp.×Dist. to mic.			0.55	**0.008**			0.36	**0.020**			0.00	0.926
		494													
*M. riparius* (*n*=12)	*T*_a_	60	Temp.	0.28	0.602	−0.31	0.715	0.24	0.329	0.93	0.081	0.02	0.125	0.02	0.495
	+2°C	62	Dist. to mic.			−6.24	0.374			7.80	0.122			0.04	0.902
	+4°C	60	Temp.×Dist. to mic.			0.33	0.319			−0.36	0.123			0.00	0.842
		182													
*M. oxyotus* (*n*=5)	*T*_a_	31	Temp.	0.02	0.918	1.56	**0.030**	−0.40	0.238	−0.45	0.565	0.06	0.073	−0.02	0.829
	+2°C	19	Dist. to mic.			17.94	**0.028**			1.71	0.827			−0.79	0.345
	+4°C	25	Temp.×Dist. to mic.			−0.84	**0.029**			0.05	0.884			0.05	0.224
		75													
*M. nigricans*/ *elegans* (*n*=8)	*T*_a_	55	Temp.	0.26	0.224	−0.44	0.585	0.06	0.081	0.49	0.464	0.03	0.081	0.01	0.867
	+2°C	49	Dist. to mic.			−7.15	0.399			6.24	0.312			−0.01	0.985
	+4°C	43	Temp.×Dist. to mic.			0.37	0.347			−0.13	0.641			0.01	0.652
		147													
*E. brasiliensis* (*n*=6)	*T*_a_	36	Temp.	−1.55	0.223	−2.92	0.318	−1.21	**0.043**	−0.74	0.544	0.04	0.319	0.05	0.624
	+2°C	44	Dist. to mic.			−18.29	0.587			4.32	0.754			0.01	0.997
	+4°C	24	Temp.×Dist. to mic.			1.01	0.513			−0.01	0.983			0.02	0.800
		104													

The table provides estimates (Est.) and *P*-values from two models: M1, a random slope model with temperature as the sole explanatory variable, and M2, a random slope model that includes the interaction between temperature and distance to the microphone as explanatory variables. Numbers in bold represent significant effects. *T*_a_, ambient temperature.

### Statistical analyses

#### The effect of temperature on echolocation calls

All statistical analyses were conducted using R version 4.0.5. To determine whether bats adapt call parameters to increased ambient temperatures, we compared two linear mixed-effects models using the R-package lme4, version 1.1-28 (https://CRAN.R-project.org/package=lme4). We calculated random slope models per species as follows:

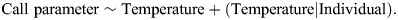
We found high inter-individual variation in call parameters in all species (Figs S1, S2 and S3), suggesting that individuals could respond differently to increasing temperatures. This variation is better captured by random slope models rather than random intercept models (Bates et al., 2015). Additionally, previous research indicates that echolocation call adjustments occur in a distance-dependent manner to obstacles (Holderied et al., 2006); to account for this potential confounder, we also constructed a model including the interaction between temperature and distance to the microphone:

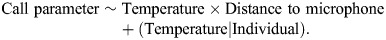
We used this model to explain species responses when there was a significant interaction effect (*P*≤0.05) between temperature and distance to the microphone on our response variable (call parameter). For detailed results of both models, see Table 1.

#### The effect of temperature on atmospheric attenuation and detection distance

To investigate whether bats adjust call parameters to weather conditions to maintain detection distance, we calculated the atmospheric attenuation (AA) of sound and the detection distance (DD) for prey based on weather conditions and call parameters. AA describes how much the level of a sound is weakened per distance, expressed in decibels per metre (dB m^−1^), and depends (in decreasing order) on call frequency, ambient temperature, relative humidity and atmospheric pressure (Goerlitz, 2018). DD is the distance over which a bat can detect an object; for example, a prey item. According to the sonar equation (Møhl, 1988), DD depends on the emitted apparent source level (aSL), AA, the sound reflectivity of the object (target strength, TS) and the bat's hearing threshold. Therefore, AA is a function of call peak frequency, temperature and RH; and DD in turn is a function of AA, aSL, prey target strength and hearing threshold (sonar equation; Møhl, 1988).

TS is strongly influenced by prey size (de Framond et al., 2023b), its orientation towards the bat (Sümer et al., 2009; Waters et al., 1995) and surface properties (Neil et al., 2020a; Simon et al., 2023). In general, neotropical vespertilionid species prefer soft prey such as nocturnal lepidoptera (moths) from a wide range of sizes (Aguirre et al., 2003; Ingala et al., 2021). To account for this variation, we set three different values of TS according to different sizes of prey: small (TS=−30 dB), medium (TS=−20 dB) and large (TS=−10 dB), at a reference distance of 10 cm (Møhl, 1988; Surlykke et al., 1999; Surlykke and Kalko, 2008; Waters et al., 1995). Note that these are just approximate values, given the various factors influencing TS (Neil et al., 2020a,b). We set the hearing threshold at 20 dB SPL to account for noise and behavioural reaction thresholds (Boonman et al., 2013).

To separate the effect of changing temperature and changing call parameters, we used two models to compare a bat that does not change its call parameters despite changing temperatures (constant call model) with a bat as observed in our experiments (flexible call model, i.e. using the actual call parameters recorded from the bats for different experimental temperatures). We first calculated, per individual, the mean values of peak frequency, aSL and duration at each experimental temperature (*T*_a_, *T*_a_+2°C, *T*_a_+4°C). In the constant call model, we only used the mean call parameters measured at *T*_a_ to calculate AA and DD at all three experimental temperatures; thus, we assume these bats do not adjust call parameters with increasing temperatures. In the flexible call model, we used the actual call parameters per temperature; thus, we included potential call adjustments as temperature increases. To include the effect of changing call duration on DD, we lowered the bat's hearing threshold by 6 dB for every doubling of duration (Luo et al., 2015; for short calls <∼2 ms) as per Eqn 1:
(1)

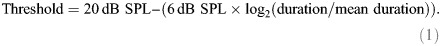
By comparing AA between the constant call and the flexible call models, we tested whether bats counteracted a potential temperature-induced increase in AA by lowering call frequency. Also, by comparing DD between the two models, we tested whether the bats counteracted a potential temperature-induced decrease in DD by adjusting call parameters, i.e. whether they maintained DD when facing warming conditions.

Using the AA and DD data for both models, we calculated the interaction effects to determine whether changes in AA and DD over temperature differed between the constant call and the flexible call models:

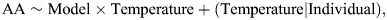


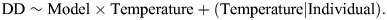


The interaction effects allowed us to quantify differences in AA or DD between constant call and flexible call models with increasing temperatures. To assess whether the distance to the microphone influences these parameters, we also performed models that included the interaction of temperature and distance to the microphone:

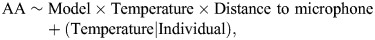


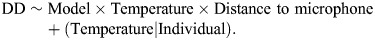


The response variables (AA and DD) were evaluated in relation to temperature and model as fixed factors, with individuals included as random effects. The factor Model comprises two categories: constant call and flexible call models. The results of these models could be interpreted as follows: (1) a significant effect of the factor Model would suggest that mean values of AA and/or DD differ between constant call and flexible call models. (2) A significant effect of Temperature would be interpreted as changing AA and/or DD values across temperatures. (3) A significant effect of the interaction (Model×Temperature) on AA and/or DD would suggest that bats respond differently to increasing temperatures between the constant call and flexible call models. (4) A significant effect of the interaction Model×Temperature×Distance to the microphone would suggest that bats respond differently to increasing temperatures between the models in a distance-dependent manner.

#### The effect of RH on AA and DD

Finally, as AA is determined by temperature and RH in a non-linear way, we estimated the effect of lower relative humidity on AA and DD at the same increasing temperature values used in our experiments. Because RH is much harder to manipulate in an open-air setup in the field, we modelled changes in AA for bats in each studied species, and we fixed RH at 50%, 75% and 100%. We tested the effect of different scenarios of RH based on the bat echolocation call parameters recorded at *T*_a_ (constant call model).

Using the constant call model, we calculated AA for each combination of temperature and RH, and then evaluated the interaction effects to determine whether changes in AA over temperature differed between the three fixed scenarios of relative humidity (RHfix). We calculated the interaction effects as follows:

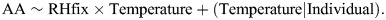
As DD is dependent on AA values, we also calculated changes in DD over temperature between the three fixed scenarios of relative humidity (RHfix) as follows:

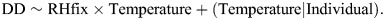
The interaction effects allowed us to quantify differences in AA or DD with increasing temperature at low, medium and high humidity scenarios (50%, 75% and 100%, respectively). The response variables were evaluated in relation to temperature and humidity as fixed factors, with individuals included as random effects. A significant effect of the factor RHfix would suggest that mean values of AA or DD differ between the simulated scenarios of RH.

## RESULTS

### The effect of temperature on echolocation calls

Our *a priori* expectation was that bats would adjust their echolocation parameters in response to increasing temperatures. However, the results presented herein show that this response was not straightforward and was often not aligned with our predictions. Furthermore, not all species responded in the same way. In some cases, the response was driven not only by temperature alone but also by the interaction between temperature and distance to the microphone. Even within species, some call parameters changed with increasing temperatures, while others did not. This variability highlights the importance of interpreting the results by species and call parameter, rather than expecting a uniform response across taxa.

**Fig. 2.**
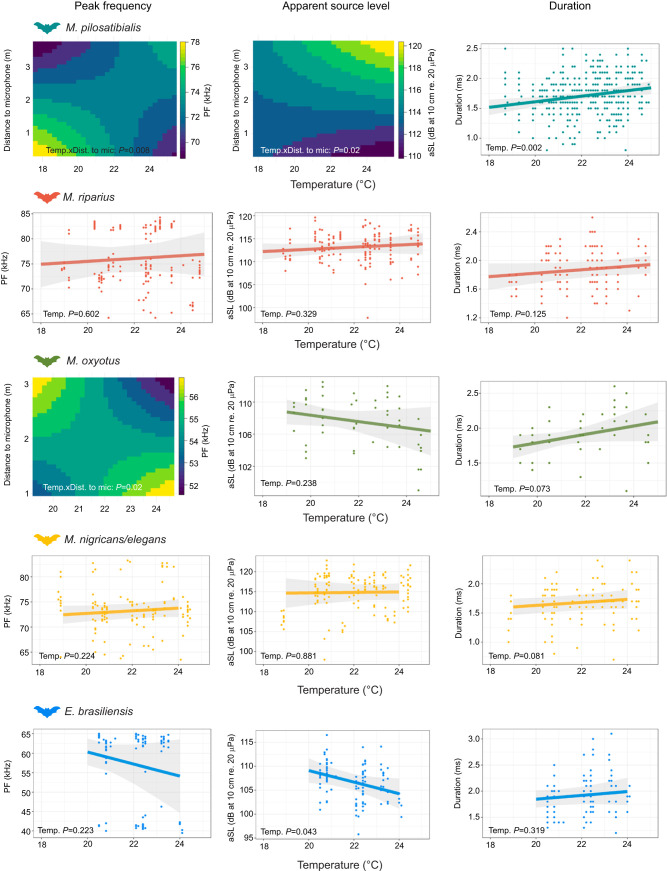
**Call parameters as a function of increasing ambient temperature (*T*_a_) per bat species (or species group).** Call parameters peak frequency (PF), apparent source level (aSL) and duration are shown for *Myotis pilosatibialis*, *Myotis riparius*, *Myotis oxyotus*, *Myotis nigricans*/*elegans* and *Eptesicus brasiliensis*. *P*-values were obtained from random slope models to test whether call parameters change with temperature. Heatmaps are shown for species where both the distance to the microphone and temperature significantly influenced the call parameter. See Table 1 for detailed data, including the number of calls used per species.

To test whether bats adjust their calls to increasing temperatures depending on the distance to a target, we included temperature and the interaction between temperature and distance to the microphone as explanatory variables in our models. The interaction significantly affected peak frequency in *M. pilosatibialis* and *M. oxyotus* (*P*=0.008 and *P*=0.029, respectively; Table 1, Fig. 2). In *M. pilosatibialis*, peak frequency was highest (∼78 kHz) when bats were recorded close to the microphone at low temperatures, or far from the microphone at high temperatures. Conversely, peak frequency decreased to around 68 kHz when temperature increased at short distances or decreased at long distances (Fig. 2). In *M. oxyotus*, the opposite pattern was observed, with peak frequency differences of about 8 kHz across the interaction gradient (Fig. 2). No significant effects were detected for the other species.

Similarly, the interaction between temperature and distance to the microphone significantly influenced apparent source level in *M. pilosatibialis* (*P*=0.02; Table 1). Specifically, aSL increased with rising temperatures when bats were recorded far from the microphone (from ∼114 to 121 dB at 10 cm re. 20 µPa), but decreased (from ∼114 to 110 dB at 10 cm re. 20 µPa) when bats were recorded close to the microphone (Fig. 2). In *E. brasiliensis*, only temperature had a significant effect, with aSL decreasing as temperature increased (*P*=0.043). For all other species, neither temperature nor its interaction with distance to the microphone had a significant effect on aSL.

Finally, the interaction between temperature and distance to the microphone did not significantly influence call duration in any of the species. Temperature alone also had no effect on duration in most species, except for *M. pilosatibialis*, where call duration increased with temperature (*P*=0.002; Fig. 2, Table 1). Average call duration at *T*_a_ (∼19°C) was 1.5 ms, whereas at higher temperatures (∼24°C) it increased to 1.9 ms.

### The effect of temperature on AA

**Fig. 3.**
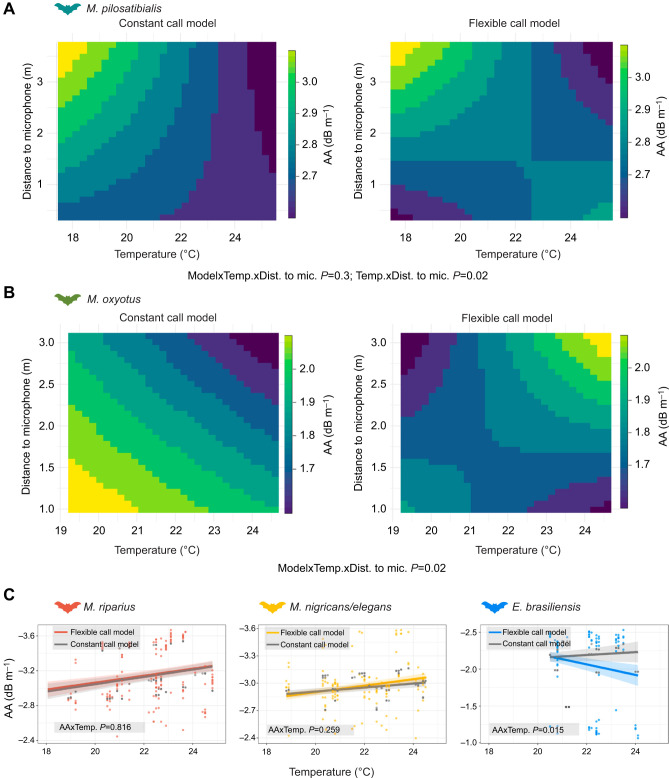
**Atmospheric attenuation (AA) as a function of increasing temperature for both measured constant call and flexible call models.** (A) *Myotis pilosatibilis* call parameters showed a significant interaction between temperature and distance to the microphone, with no difference between constant call and flexible call models. (B) *Myotis oxyotus* call parameters showed a significant interaction between temperature and distance to the microphone, differently for each model. (C) Dots show individual data, calculated either for actual call parameters and weather conditions (flexible call model: coloured) or for call parameters at *T*_a_ and actual weather data (constant call model: grey). Lines are model results with the 95% confidence interval (shaded region). *P*-values in each panel indicate whether the slope of AA over increasing temperature differs between constant call and flexible call models. Samples are based on individual calls. See Table 1 for the number of calls used per species**.**

To quantify the combined effects of call frequency and weather conditions on AA, we compared two models: a constant call model with no call adjustments and a flexible call model with actual calls emitted by bats at each experimental temperature and distance to the microphone, which considers potential call adjustments (Fig. 3, Table 2). For *M. pilosatibialis*, we found that the interaction between temperature and distance to the microphone was significant (*P*=0.02; Table 2); however, AA did not change when analysing the constant call and flexible call conditions separately (*P*=0.3). Specifically, we found that for both models, AA at short distances remained relatively constant (∼3 dB m^−1^), whereas at longer distances, AA decreased by ∼0.2 dB m^−1^ as temperatures increased (Fig. 3A). We found a significant interaction among the three explanatory variables in *M. oxyotus*: model type, temperature and distance to the microphone (*P*=0.02, Table 2). In the constant call model, we found a consistent decrease in AA with temperature and distance to the microphone (from ∼2.06 to 1.91 dB m^−1^; Fig. 3B). In the flexible call model, we found that at short distances, AA decreased by 0.25 dB m^−1^ with an increase in temperature from 19 to 25°C; in contrast, at longer distances, AA increased by 0.4 dB m^−1^ with increasing temperatures (Fig. 3B). Lastly, in *E. brasiliensis*, we found different AA patterns between the models (Fig. 3C; model×temperature: *P*=0.015). In the flexible call model, AA decreased from 2.2 to 1.9 dB m^−1^ with increasing temperatures, while it remained constant at 2.2 dB m^−1^ in the constant call model. For the other species, temperature and distance to the microphone did not have a significant effect on AA (Table 2).

**Table 2.** Results of the random slope model for predicting atmospheric attenuation (AA) with temperature as the sole explanatory variable (M1) or the model that includes the interaction between temperature and distance to the microphone as explanatory variables (M2)

Species	Variable	AA (dB m^−1^)			
		M1		M2	
		Est.	*P*	Est.	*P*
*M. pilosatibialis*	Model	0.03	0.868	0.71	0.191
	Temp.	−0.01	0.142	0.03	0.10
	Model×Temp.	−0.0	0.775	−0.03	0.178
	Dist. to mic.			0.50	**0.01**
	Model×Dist. to mic.			−0.35	1.88
	Temp.×Dist. to mic.			−0.02	**0.02**
	Model×Temp.×Dist. to mic.			0.02	0.187
*M. riparius*	Model	0.07	0.767	−0.01	0.989
	Temp.	−0.04	**0.026**	−0.02	0.533
	Model×Temp.	−0.00	0.816	0.00	0.973
	Dist. to mic.			0.19	0.560
	Model×Dist. to mic.			0.04	0.926
	Temp.×Dist. to mic.			−0.1	0.518
	Model×Temp.×Dist. to mic.			−0.00	0.924
*M. oxyotus*	Model	0.29	0.287	−1.77	0.058
	Temp.	0.01	0.321	−0.09	**0.007**
	Model×Temp.	−0.02	0.178	0.08	0.062
	Dist. to mic.			−1.12	**0.002**
	Model×Dist. to mic.			1.14	**0.023**
	Temp.×Dist. to mic.			0.05	**0.002**
	Model×Temp.×Dist. to mic.			−0.05	**0.020**
*M. nigricans/elegans*	Model	−0.27	0.291	0.61	0.536
	Temp.	−0.03	**0.001**	0.01	0.769
	Model×Temp.	0.01	0.259	−0.03	0.499
	Dist. to mic.			0.46	0.182
	Model×Dist. to mic.			−0.45	0.343
	Temp.×Dist. to mic.			−0.02	0.148
	Model×Temp.×Dist. to mic.			0.02	0.306
*E. brasiliensis*	Model	2.14	**0.022**	6.69	**0.041**
	Temp.	0.09	**0.044**	0.22	**0.042**
	Model×Temp.	−0.10	**0.015**	−0.33	**0.027**
	Dist. to mic.			1.78	0.165
	Model×Dist. to mic.			−2.85	0.107
	Temp.×Dist. to mic.			−0.09	0.121
	Model×Temp.×Dist. to mic.			0.14	0.082

In the variable list, ‘model’ corresponds to an effect by using either the constant call or flexible call model.

### The effect of temperature on DD

**Fig. 4.**
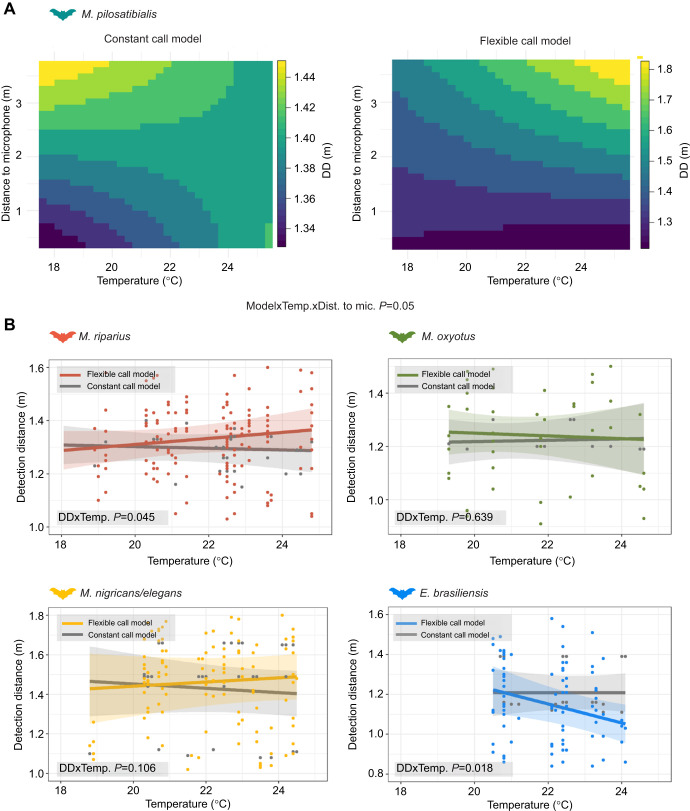
**Detection distance (DD) as a function of increasing temperature for both constant call and flexible call models.** (A) Heatmap for *M. pilosatibialis* for which the interaction of temperature×distance to the microphone was significant. (B) Dots show individual data, calculated either for actual call parameters and weather conditions (flexible call model: coloured) or for call parameters at *T*_a_ and actual weather data (constant call model: grey). Lines are model results with the 95% confidence interval (shaded region). *P*-values in each panel indicate whether the slope of AA over increasing temperature differs between constant call and flexible call models. Samples are based on individual calls. See Table 1 for the number of calls used per species**.**

To quantify the contribution of the combined changes in call parameters and weather conditions on prey DD, we compared the DD for prey of bats in the constant call model with the DD of the bats in the flexible call model (Fig. 4, Table 3). DD was constant across temperatures for most *Myotis* species in both models (Fig. 4, Table 3), except for *M. riparius*, where DD slopes differed over temperature between models (*P*=0.045), with an increase of 0.1 m in DD for the flexible model. As for AA, the slopes of DD differed over temperature between models in *E. brasiliensis* (*P*=0.018, Table 2). In *M. pilosatibialis*, we found a significant interaction among the three explanatory variables, model, temperature and distance to the microphone (*P*=0.047, Table 3). For the constant call model, we found that DD increased for shorter distances with increasing temperatures; the opposite was observed for longer distances, where DD decreased by 0.05 m (Fig. 4A). Temperature and distance to the microphone did not have a significant effect on DD for the other species (Table 3). In the flexible call model, we found that DD remained constant at shorter distances with an increase in temperature, and at longer distances DD increased with increasing temperature by 0.33 m (Fig. 4A). For the bats in the flexible call model, DD decreased with increasing temperature from 1.22 to 1.03 m (Fig. 4B and Table 3); in contrast, we did not see an effect of temperature on DD in the constant call model. These results for the effect on DD were calculated for medium-sized prey (20 mm^2^), but increasing or decreasing prey size by 10 mm^2^ resulted in a corresponding change of DD by ∼0.5 m (Table S2).

**Table 3.** Results of the random slope model for predicting detection distance (DD) with temperature as the sole explanatory variable (M1) or the model that includes the interaction between temperature and distance to the microphone as explanatory variables (M2)

Species	Variable	DD (m)			
		M1		M2	
		Est.	*P*	Est.	*P*
*M. pilosatibialis*	Model	−0.22	0.160	0.43	0.397
	Temp.	0.00	0.770	0.01	0.456
	Model×Temp.	0.01	0.085	−0.03	0.226
	Dist. to mic.			0.13	0.459
	Model×Dist. to mic.			−0.37	0.133
	Temp.×Dist. to mic.			−0.01	0.513
	Model×Temp.×Dist. to mic.			0.02	**0.047**
*M. riparius*	Model	−0.28	0.075	−0.54	0.390
	Temp.	−0.00	0.719	0.01	0.579
	Model×Temp.	0.01	**0.045**	0.02	0.503
	Dist. to mic.			0.17	0.440
	Model×Dist. to mic.			0.07	0.820
	Temp.×Dist. to mic.			−0.01	0.355
	Model×Temp.×Dist. to mic.			−0.00	0.966
*M. oxyotus*	Model	0.19	0.607	0.96	0.404
	Temp.	0.00	0.875	0.02	0.696
	Model×Temp.	−0.01	0.639	−0.06	0.272
	Dist. to mic.			0.15	0.734
	Model×Dist. to mic.			0.42	0.491
	Temp.×Dist. to mic.			0.01	0.754
	Model×Temp.×Dist. to mic.			0.03	0.322
*M. nigricans/elegans*	Model	−0.44	0.132	−0.13	0.901
	Temp.	−0.01	0.453	0.02	0.604
	Model×Temp.	0.02	0.106	−0.02	0.731
	Dist. to mic.			0.30	0.393
	Model×Dist. to mic.			−0.26	0.590
	Temp.×Dist. to mic.			−0.01	0.383
	Model×Temp.×Dist. to mic.			0.02	0.291
*E. brasiliensis*	Model	0.99	**0.003**	1.91	0.168
	Temp.	−0.00	0.983	0.04	0.389
	Model×Temp.	−0.05	**0.002**	−0.11	0.089
	Dist. to mic.			0.46	0.395
	Model×Dist. to mic.			−0.81	0.280
	Temp.×Dist. to mic.			−0.02	0.298
	Model×Temp.×Dist. to mic.			0.05	0.166

In the variable list, ‘model’ corresponds to an effect by using either the constant call or flexible call model. Numbers in bold represent significant effects.

### The effect of RH on AA and DD

**Fig. 5.**
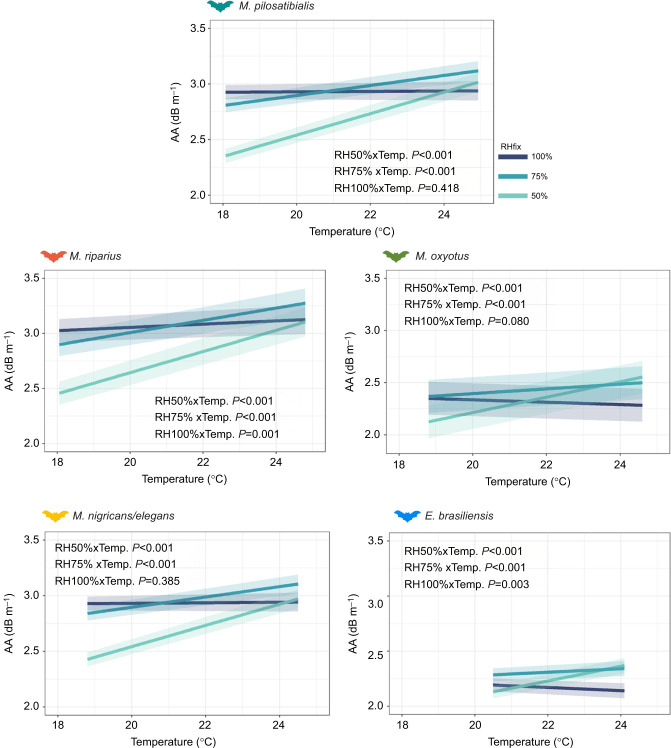
**Variation in AA per species with increasing temperature.** Relative humidity (RH) values were fixed at 100%, 75% and 50% (RHfix) to show potential changes between drier and more humid conditions. Results are based on constant echolocation call parameters (constant call model). *P*-values in each panel show whether the slope of AA over increasing temperature differs between low and medium RH scenarios compared with a high RH setting. See Table 1 for the number of calls used per species**.**

By evaluating the effect of different RH scenarios on AA and DD, we found that all species would experience significantly different patterns of AA and DD (*P*<0.001) across low, medium and high humidity conditions, with particularly distinct differences under the low RH (50%) scenario (Fig. 5; Fig. S4). At 50% RH, AA increased in all *Myotis* species by at least 0.5 dB m^−1^ and up to 0.8 dB m^−1^ with increasing temperatures (Table S1). At medium RH (75%), the increase of AA ranged from 0.2 dB m^−1^ to 0.4 dB m^−1^ (Table S1). Likewise, DD may decrease by up to 14 cm in *M. pilosatibialis* and 9 cm in *M. nigricans*/*elegans* at 50% RH (Table S2). At medium RH (75%), most species showed a reduction in DD ranging from 3 to 5 cm. For all species, both AA and DD remained constant at high RH (100%; Tables S1 and S2).

## DISCUSSION

Echolocation is highly dynamic, and bats adapt their calls constantly to changing conditions. Thus, we expected adaptive changes in the echolocation calls from our studied species to counteract the predicted reduction of prey DD caused by increasing AA because of changing *T*_a_. We found an increment in AA with increasing *T*_a_ for *M. pilosatibialis*, which would lead to a theoretical reduction in DD of prey in the constant call model, especially at longer distances. However, we observed an increase in DD in the flexible call model for longer distances, which suggests that this species may be compensating for the effect of temperature on AA by increasing its call source level and duration. Indeed, and aligning with our prediction, *M. pilosatibilis* increased its aSL when echolocating over greater distances, along with a general increase in call duration. This was the most significant finding in our study, as this species was the only one to show changes in echolocation parameters in a predictable manner. These behavioural adaptations may underlie the observed increase in DD at higher temperatures and greater distances despite the increased values of AA. *Myotis oxyotu*s may also be compensating for an increase in AA due to increasing temperatures at longer distances by reducing their call peak frequency, although seemingly not enough to increase DD. The findings in both species together indicate that the anticipated rise in *T*_a_ due to climate change could impair prey detection over longer distances in these two species. However, they may be capable of adapting by flexibly adjusting their call parameters to reduce the negative effects of AA.

We also observed responses that did not match our prediction. The most notable example is *E. brasiliensis*, where we found a decrease in aSL with increasing temperature and no significant differences in any of the other call parameters. In this species, we also observed a decrease in AA in the flexible call model, which was probably due to two factors: (1) a reduction in peak frequency from 60.3 kHz at 20°C to 54.0 kHz at 24°C, though non-significant (Fig. 2) and (2) the effect of increasing temperatures slightly reducing AA at call frequencies of 55–60 kHz under the prevalent weather conditions (20–24°C, >60% RH; Goerlitz, 2018). Furthermore, despite the reduction in AA, this species also experienced a decrease in DD, contrary to our expectations. This can be explained by the reduction in aSL from the lowest temperature to the highest (106 dB to 101 dB, respectively), which may have affected DD in the flexible model.

While we found that an increase in temperature affected AA in some species, for others we did not detect an effect. One reason for this lack of change in AA might be that, for these species, the variation in temperature experienced during the experiments was too low to have an effect. For example, in a temperate habitat with strong variations in temperature and humidity (differences >16°C and >40% RH, respectively), AA increased by 0.7 dB m^−1^ (de Framond et al., 2023a,b). In contrast, in our study, variation in weather conditions was considerably smaller (<7°C and <25% RH), causing AA to change by only 0.1–0.3 dB m^−1^, resulting in a two-way echo-level reduction of only 0.3–0.6 dB over the modelled prey DD of ∼1.5 m. Also, our findings suggest that the effect of rising temperatures on AA for *M. pilosatibialis* and *M. oxyotu*s, with its concomitant effect on DD, was relatively minor despite being significant. Our experimental increase of temperature by 2 and 4°C probably had a minimal effect on AA and prey DD. Although some species seemed to be attempting to adjust their call parameters, the anticipated increases in average temperature due to climate change will probably not significantly affect the sensory range of the bat species studied here, at least under the prevailing RH levels at our study site.

RH also affects AA, which in turn impacts the maximum DD achieved by bat echolocation (Goerlitz, 2018). Tropical forests in Central America are forecasted to suffer a reduction in precipitation as a result of climate change (Lyra et al., 2017), with significant drying trends in southern Costa Rica (Hidalgo et al., 2017). At the prevailing temperature conditions of our study site, increasing *T*_a_ will have a stronger increasing effect on AA when RH is lower and for call frequencies around 55–75 kHz (Goerlitz, 2018). When considering different values of RH, our results suggest that AA may have a stronger effect in drier conditions (Fig. 5), consequently decreasing prey DD (Fig. S4). These findings align with predictions of other theoretical and empirical studies (Goerlitz, 2018; Lawrence and Simmons, 1982; Snell-Rood, 2012), suggesting that Neotropical montane bat species with high-frequency echolocation calls (>70 kHz) might suffer from reduced prey detection ability as mountain ecosystems become drier and warmer. Further studies will need to address whether bats experience stronger changes in AA under these conditions and whether they will behaviourally adjust calls in response to drier conditions.

We found that bats adjusted echolocation calls in response to temperature changes but in a distance-dependent manner. For example, while we found that changes in temperature prompted changes in peak frequency and aSL in *M. pilosatibialis* and *M. oxyotus*, the adjustments in these parameters were also explained by the distance at which they emitted the call. In *M. pilosatibialis*, a significant decrease in peak frequency occurred when the temperature increased at low distances or when the distance to the microphone increased, particularly at low temperatures; the opposite trend was observed in *M. oxyotus*. Previous studies have found that bats can adjust call design, namely duration, and bandwidth, depending on the distance to objects; these adjustments result in closer objects being perceived with greater accuracy (Holderied et al., 2006). At the moment, we do not have sufficient information to explain the observed changes in call parameters caused by the combined effect of temperature with distance. Exploring this topic further would help us better understand how anticipated temperature changes might impact object and prey detection in these and other bat species.

We expected bats to increase their call source level in response to increasing AA. Only *E. brasiliensis* and *M. pilosatibialis* seem to be capable of adjusting this parameter. While echolocation during flight poses no additional energetic costs (Speakman and Racey, 1991; Voigt and Lewanzik, 2012), this might not be the case for very high-intensity calls (>∼110 dB SPL at 10 cm; Currie et al., 2020). Hence, bats may experience physiological constraints to increase call levels beyond a certain threshold. Similarly, bats may experience trade-offs when (strongly) reducing call frequency. By lowering call frequency, bats can increase DD but, in parallel, confine prey detection to larger items because lower frequencies are less reflected on smaller objects (de Framond et al., 2023b; Jung et al., 2014). Excluding a part of the potential prey spectrum by reducing call frequency could reduce a bat's foraging success. Lowering call frequency to maintain prey DD while excluding smaller prey items is a trade-off that is most likely context specific. For example, individuals that normally consume larger prey items would not suffer major losses when decreasing call frequency, whereas those that typically consume smaller prey would probably not be able to trade off a large portion of available prey for an increase in DD. This might explain why changes in peak frequency were not widely observed in our study, in contrast to what we originally predicted.

While most studies to date have investigated how bats may adapt frequency and source level to deal with changes in AA and other auditory challenges (de Framond et al., 2023b; Snell-Rood, 2012; Surlykke and Kalko, 2008), only a few have considered the effect of changing call duration on sound perception and DD (but see Chaverri and Quirós, 2017; Luo et al., 2014; Schmidt and Thaller, 1994). Our study provides additional support that *M. pilosatibialis* may adjust call duration as a potential mechanism to improve signal detection. Increasing call duration improves signal detectability by about 6 dB per doubling of duration for short calls (Luo et al., 2014). Bats may increase call duration in noisy environments (Corcoran and Moss, 2017; Luo et al., 2015; Tressler and Smotherman, 2009), and our results suggest that bats may use the same mechanism to counteract reduced echo levels to improve DD. However, bats increased call duration by ∼0.1–0.4 ms for average call durations of ∼1.5–2.0 ms, i.e. by a factor of ∼1.05- to 1.27-fold, resulting in an increase in signal detectability of 0.2–0.9 dB. Given these small effect sizes, more studies will be needed to evaluate the relevance of call duration for improving signal detectability and its dependence on other constraints, for example, whether changing frequency and source level might have interacting effects. It is important to note that increasing call duration may lead to greater temporal overlap with returning echoes, as bats usually shorten their calls to minimize this effect. In fact, an additional 1 ms of signal duration extends the overlap zone by approximately 17 cm (Holderied et al., 2006). Thus, although increasing call duration may enhance detectability, it could also entail trade-offs by increasing the likelihood of echo interference and associated energetic or perceptual costs.

To our knowledge, this is the first experimental assessment of short-term adjustments of echolocation calls to experimentally raised *T*_a_ in the Neotropical region, providing the first data about a scarcely studied topic (Festa et al., 2022). Our results suggest that the average effect of warming on DD seems to be small for close-range prey detection, probably precluding the need for call adjustments in some bat species and under specific weather conditions. Nevertheless, future studies are needed to understand how call types, call function, behavioural context and ecology interact and affect sound perception in a wider range of species and weather conditions and how bats deal with changes potentially challenging their perception. For example, in response to changing weather conditions, two species of molossid bats did not change their frequency-modulated calls that are used for close-range object detection and which are similar to the calls of the species in our study. In contrast, they adjusted their lower-frequency, constant-frequency calls that are used for long-range object detection (Chaverri and Quirós, 2017). Further research is needed to ascertain whether other bat species will be affected by changing weather and climatic conditions and whether they will be capable of adjusting their echolocation calls, their most important sensory input.

## Supplementary Material

10.1242/jexbio.251076_sup1 Supplementary information
