# Supplementary material for: Variation in echolocation call emission of Neotropical insect-eating bats in response to shifting ambient temperatures
Source: J Exp Biol. 2025 Oct 24;228(20):jeb251076. doi: 10.1242/jeb.251076 (PMC12582406; doi:10.1242/jeb.251076)
Supplement: Supplementary information [file jexbio-228-251076-s1.pdf]

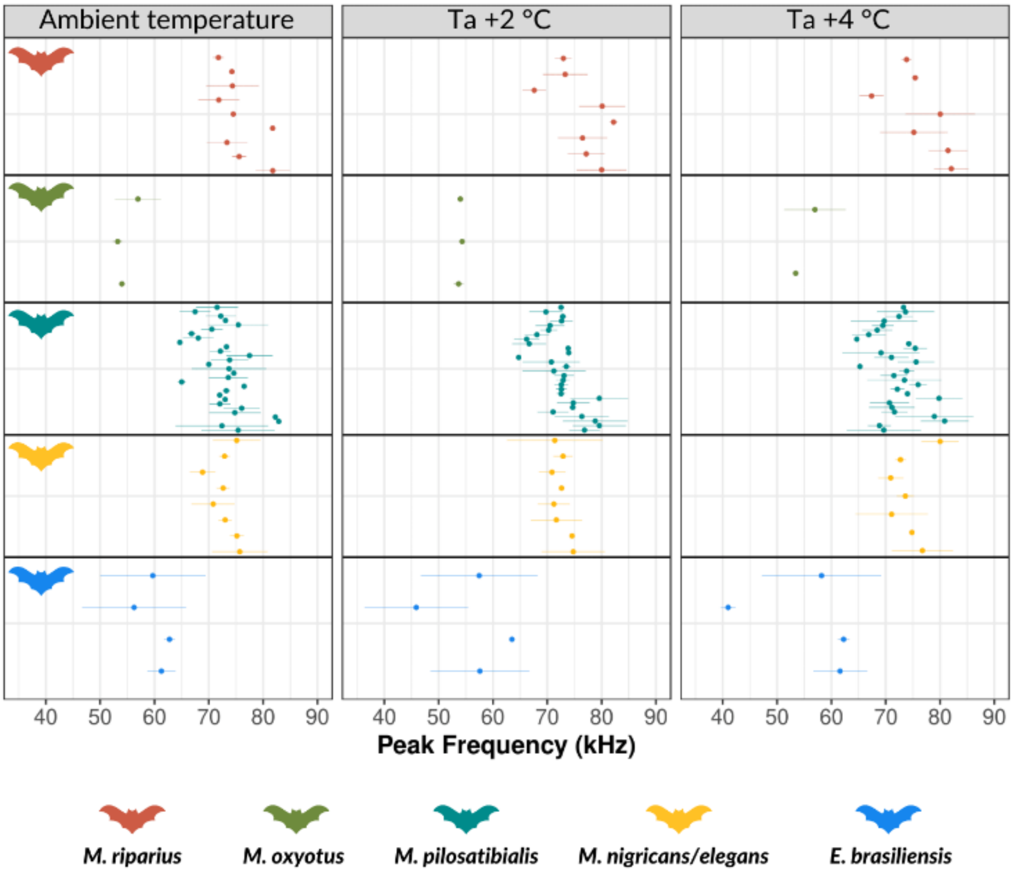

Fig. S1. Variability of call peak frequency per individual within species at different temperatures. 1) Ambient temperature, 2) increased temperature by 2°C, and 3) increased temperature by 4°C. Each dot represents one individual.

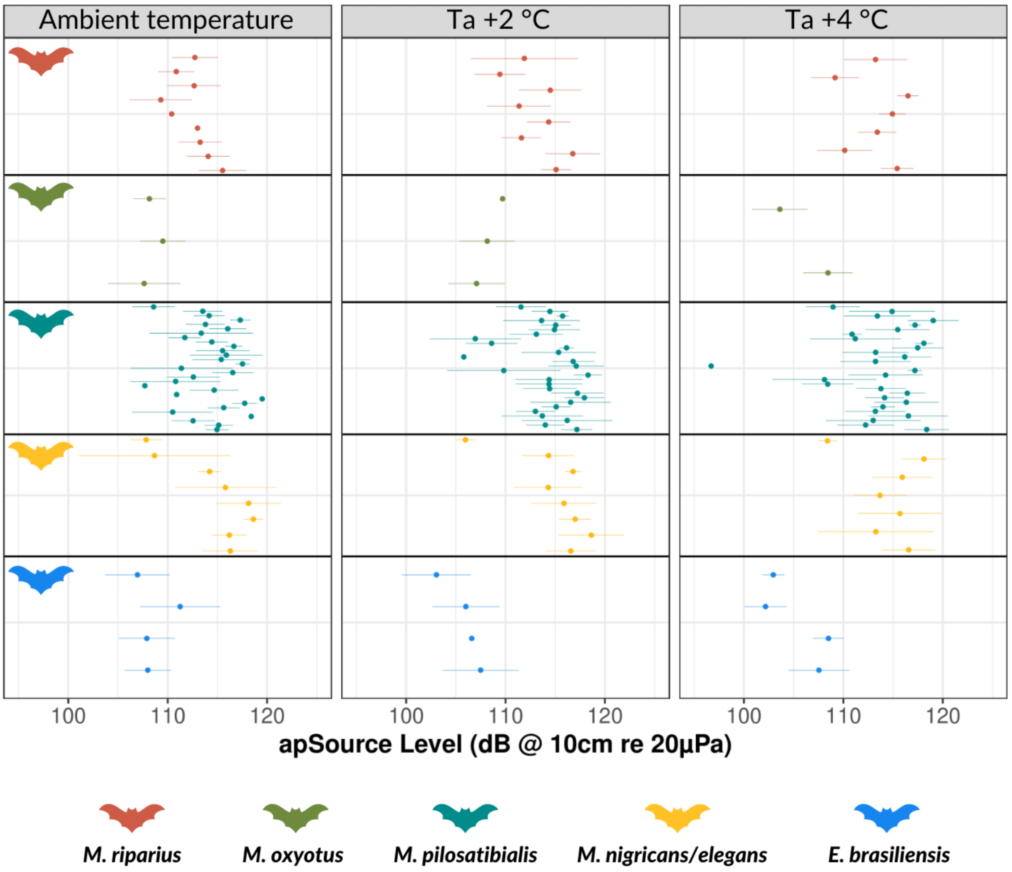

Fig. S2. Variability of call apparent source level per individual within species at different temperatures. 1) Ambient temperature, 2) increased temperature by 2°C, and 3) increased temperature by 4°C. Each dot represents one individual.

Journal of Experimental Biology • Supplementary information

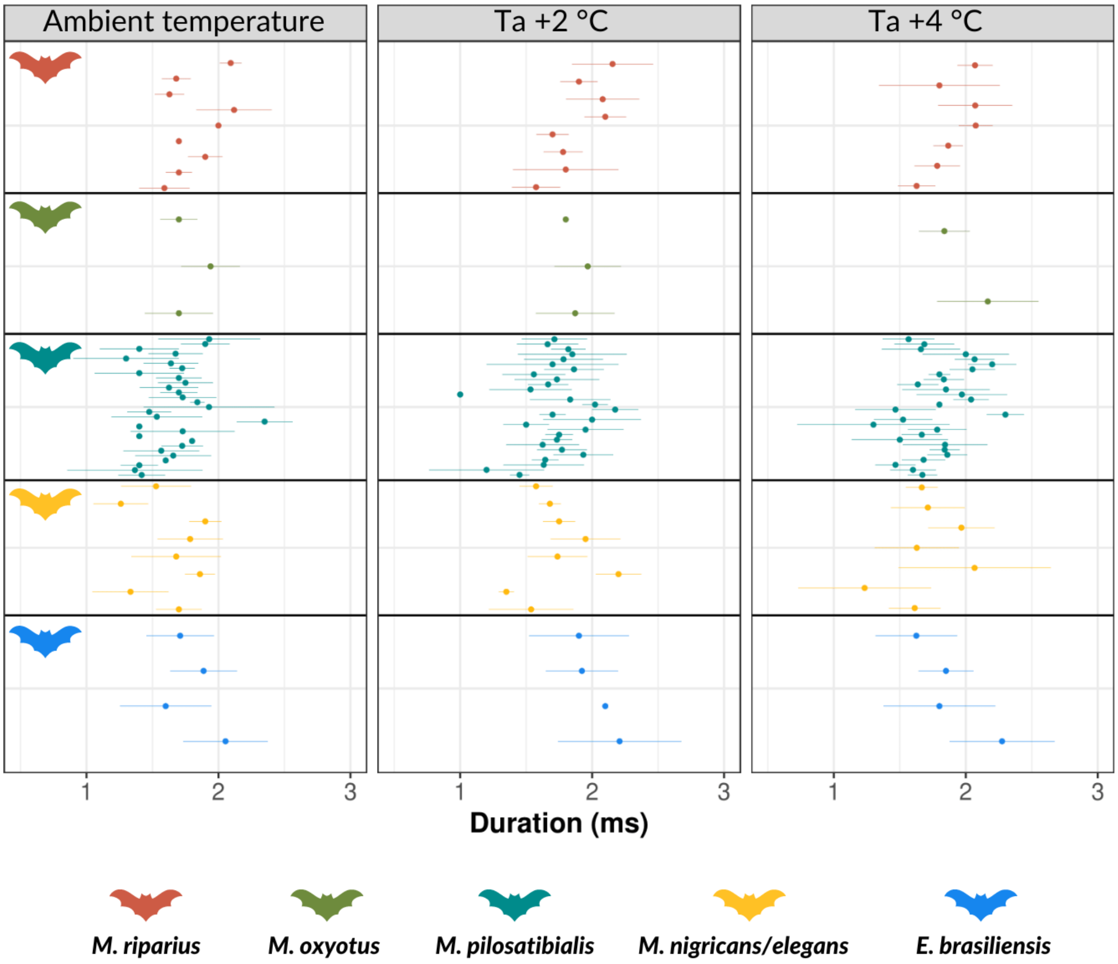

Fig. S3. Variability of call duration per individual within species at different temperatures. 1) Ambient temperature, 2) increased temperature by 2°C, and 3) increased temperature by 4°C. Each dot represents one individual.

Journal of Experimental Biology • Supplementary information

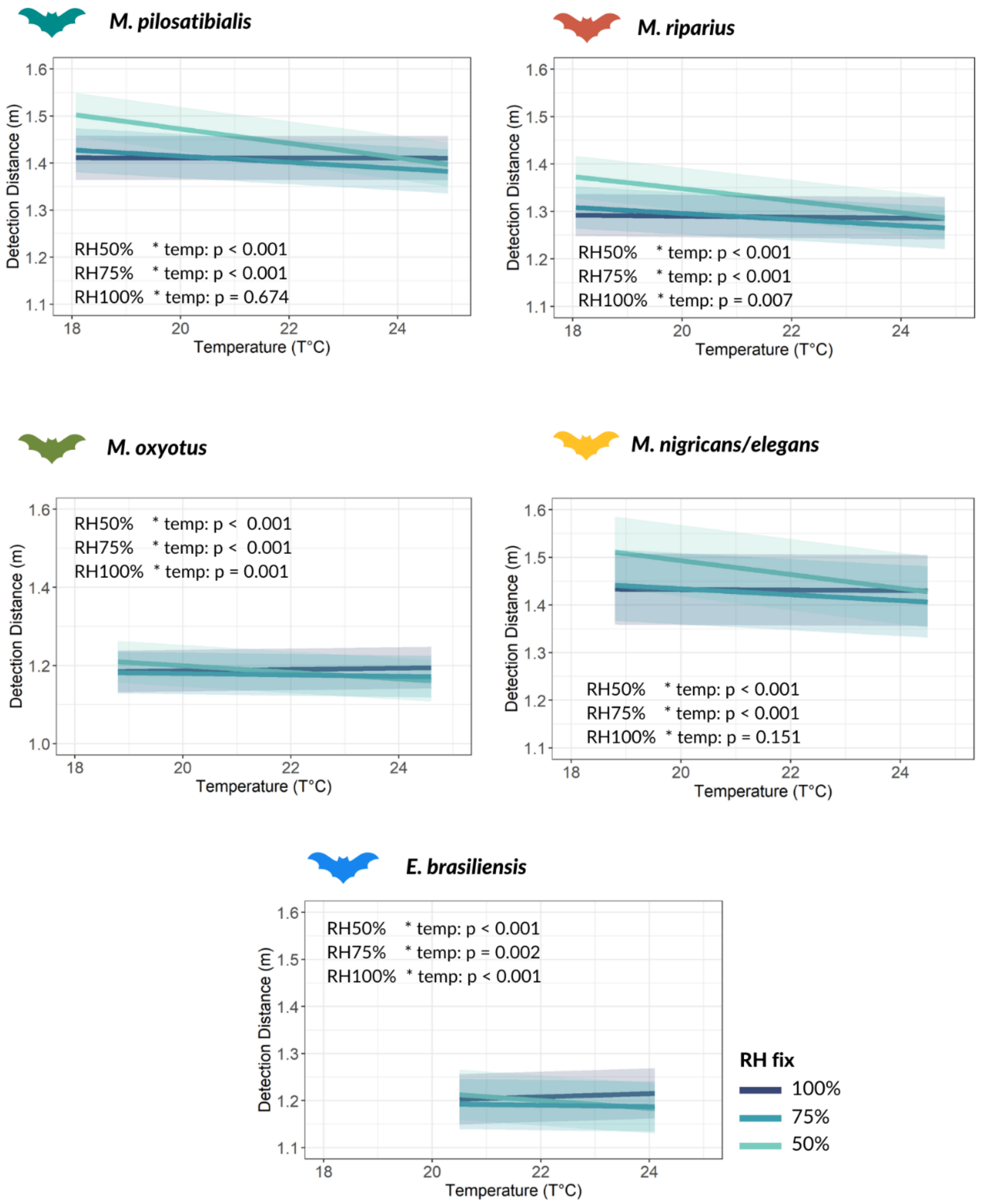

Fig. S4. Variation in detection distance per species with increasing temperatures. Relative humidity values were fixed at 100%, 75%, and 50% (RH fix) to show potential changes between drier and more humid conditions. Results are based on constant echolocation call parameters (*constant call* model). P-values in each panel show that the slope of DD over increasing temperature differs between low and medium relative humidity scenarios compared to high relative humidity settings.

Table S1. Mean values of AA for each experimental temperature at three fixed values of relative humidity (50%, 75% and 100%). Columns Δ1 and Δ2 represent the difference in AA at +2°C and +4°C, respectively, relative to the ambient temperature

		**Atmospheric Attenuation (dB/m) at fixed RH**				
	**RHfix**	**Ta°C**	**+2°C**	**+4°C**	**Δ1**	**Δ2**
M. pilosatibialis	50%	2.3	2.7	3.1	**0.4**	**0.8**
	75%	2.8	3.0	3.1	**0.2**	**0.4**
	100%	2.9	2.9	2.9	**0.0**	**0.0**
M. riparius	50%	2.4	2.7	3.1	**0.1**	**0.6**
	75%	2.9	3.1	3.3	**0.1**	**0.3**
	100%	3.0	3.0	3.1	**0.0**	**0.1**
M. oxyotus	50%	2.1	2.4	2.6	**0.3**	**0.5**
	75%	2.4	2.4	2.5	**0.1**	**0.2**
	100%	2.4	2.3	2.3	**0.0**	**-0.1**
M. nigricans/elegans	50%	2.4	2.7	3.0	**0.3**	**0.6**
	75%	2.8	3.0	3.1	**0.1**	**0.3**
	100%	2.9	2.9	2.9	**0.0**	**0.0**
E. brasiliensis	50%	2.1	2.3	2.4	**0.2**	**0.1**
	75%	2.3	2.3	2.3	**0.0**	**0.0**
	100%	2.2	2.2	2.1	**0.0**	**-0.1**

Table S2. Mean values of DD for each experimental temperature at three fixed values of relative humidity (50%, 75%, and 100%). Columns Δ1 and Δ2 represent the difference in AA at +2°C and +4°C, respectively, relative to the ambient temperature

		**Detection Distance (m) at fixed RH**				
	**RHfix**	**Ta°C**	**+2°C**	**+4°C**	**Δ1**	**Δ2**
M. pilosatibialis	50%	1.52	1.44	1.38	**-0.08**	**-0.14**
	75%	1.43	1.40	1.38	**-0.03**	**-0.05**
	100%	1.41	1.41	1.41	**0.0**	**0.0**
M. riparius	50%	1.38	1.34	1.28	**-0.04**	**-0.1**
	75%	1.31	1.29	1.26	**-0.02**	**-0.05**
	100%	1.29	1.29	1.29	**0.0**	**0.0**
M. oxyotus	50%	1.21	1.18	1.16	**-0.03**	**-0.05**
	75%	1.18	1.18	1.17	**0.0**	**-0.01**
	100%	1.18	1.19	1.19	**0.01**	**0.01**
M. nigricans/elegans	50%	1.52	1.47	1.43	**-0.05**	**-0.09**
	75%	1.44	1.42	1.41	**-0.02**	**-0.03**
	100%	1.43	1.43	1.43	**0.0**	**0.0**
E. brasiliensis	50%	1.21	1.20	1.18	**-0.01**	**-0.03**
	75%	1.19	1.19	1.19	**0.0**	**0.0**
	100%	1.20	1.21	1.22	**0.01**	**0.02**
